# Supplementary material for: Nanostructure-specific X-ray tomography reveals myelin levels, integrity and axon orientations in mouse and human nervous tissue
Source: Nat Commun. 2021 May 19;12:2941. doi: 10.1038/s41467-021-22719-7 (PMC8134484; doi:10.1038/s41467-021-22719-7)
Supplement: Supplementary file 1 — Supplementary Information [file 41467_2021_22719_MOESM1_ESM.pdf]

SUPPLEMENTARY FIGURES

Nanostructure-specific X-ray tomography reveals myelin levels, integrity and axon orientations in mouse and human nervous tissue

Marios Georgiadis[1,2,3], Aileen Schroeter[1], Zirui Gao[4,1], Manuel Guizar-Sicairos[4], Marianne Liebi[5], Christoph Leuze[3], Jennifer A. McNab[3], Aleezah Balolia[6], Jelle Veraart[2], Benjamin Ades-Aron[2], Sunglyoung Kim[2], Timothy Shepherd[2], Choong H. Lee[2], Piotr Walczak[7,8], Shirish Chodankar[9], Phillip DiGiacomo[3], Gergely David[10], Mark Augath[1], Valerio Zerbi[1], Stefan Sommer[1], Ivan Rajkovic[11], Thomas Weiss[11], Oliver Bunk[4], Lin Yang[9], Jiangyang Zhang[2], Dmitry S. Novikov[2], Michael Zeineh[3], Els Fieremans[2†], Markus Rudin[1,12†]

[1] Institute for Biomedical Engineering, ETH Zurich, Zurich, Switzerland

[2] Center for Biomedical Imaging, New York University School of Medicine, NY, USA

[3] Department of Radiology, Stanford School of Medicine, Stanford, CA, USA

[4] Swiss Light Source, Paul Scherrer Institute, Villigen, Switzerland

[5] Department of Physics, Chalmers University of Technology, Gothenburg, Sweden

[6] Department of Integrative Biology, University of Colorado Denver, CO, USA

[7] Department of Radiology, Johns Hopkins Medicine, Baltimore, MD, USA

[8] Department of Diagnostic Radiology & Nuclear Medicine, University of Maryland, MD, USA

[9] National Synchrotron Light Source II, Brookhaven National Laboratory, NY, USA

[10] Balgrist University Hospital, University of Zurich, Zurich, Switzerland

[11] Stanford Synchrotron Radiation Lightsource, SLAC National Accelerator Laboratory, CA, USA

[12] Institute of Pharmacology and Toxicology, University of Zurich, Zurich, Switzerland

[*] Corresponding author: Dr. Marios Georgiadis
e-mail: mariosg@stanford.edu

[†] These authors contributed equally to this work

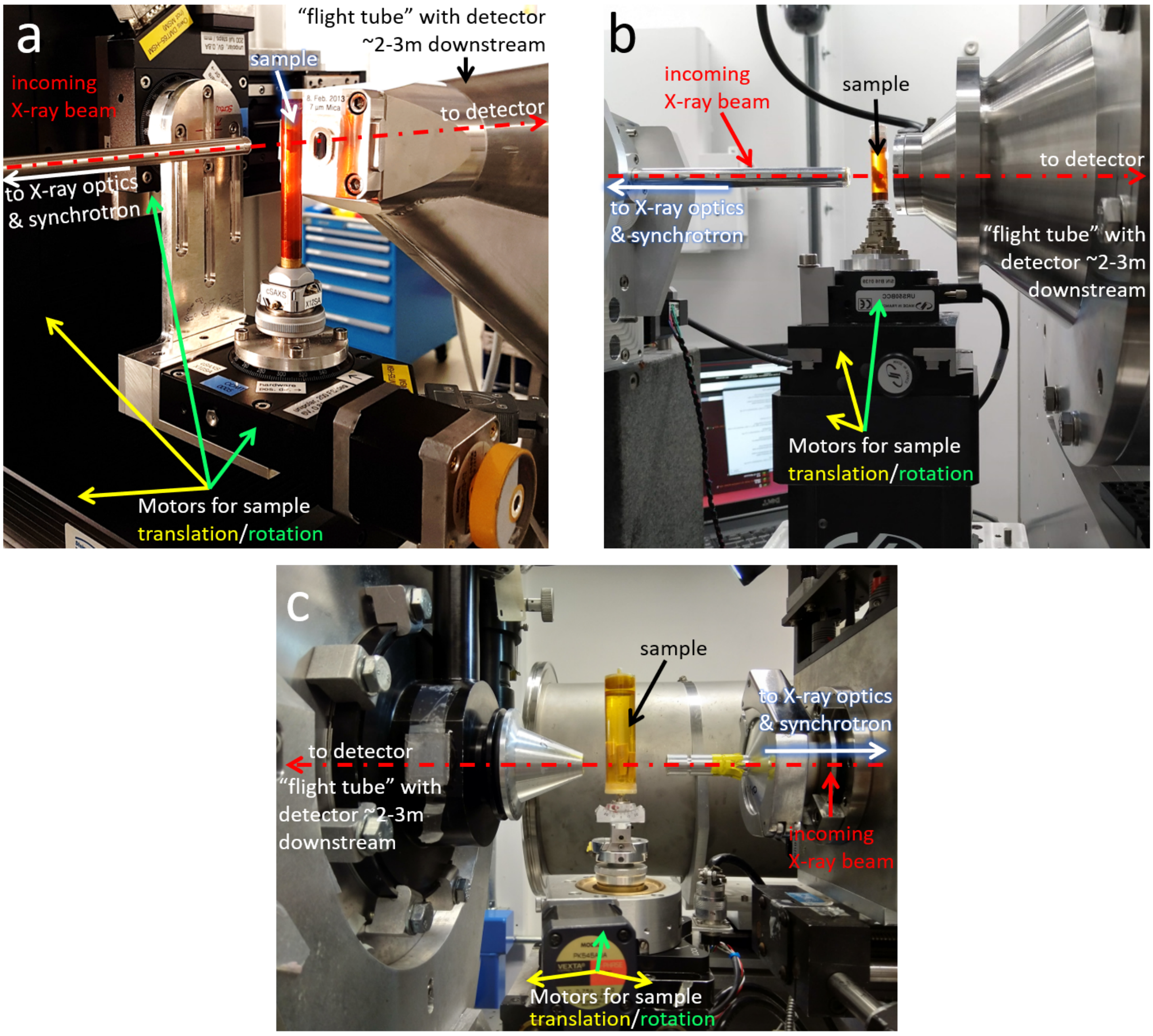

Supplementary Fig. 1. SAXS-TT setup in 3 synchrotrons.
a) SAXS-TT setup in cSAXS beamline, at the Swiss Light Source synchrotron, in the Paul Scherrer Institute, Villigen, Switzerland. **b)** SAXS-TT setup in LiX beamline,[56] at National Light Synchrotron Source II, in the Brookhaven National Laboratory, New York, USA. **c)** SAXS-TT setup in beamline 4-2, at the Stanford Synchrotron Radiation Lightsource synchrotron, in the SLAC National Accelerator Laboratory, California, USA. An undulator (cSAXS, LiX) or a wiggler (4-2) produces a very intense X-ray beam, which is focused and shaped by X-ray optics, and sent to interact with the sample (dashed red line). Motorized translation and rotation stages realize the raster scanning of the sample sitting in a Kapton tube, at multiple projections around one (b, c) or two (a) rotation axes. The photons that have interacted with the sample and are scattered at small angles travel through an evacuated "flight tube" and are collected by a photon-counting Pilatus detector[57] ~2-3m downstream. The direct beam is collected by a photodiode, which both blocks it from reaching the photon-sensitive detector and measures it to obtain transmission information.

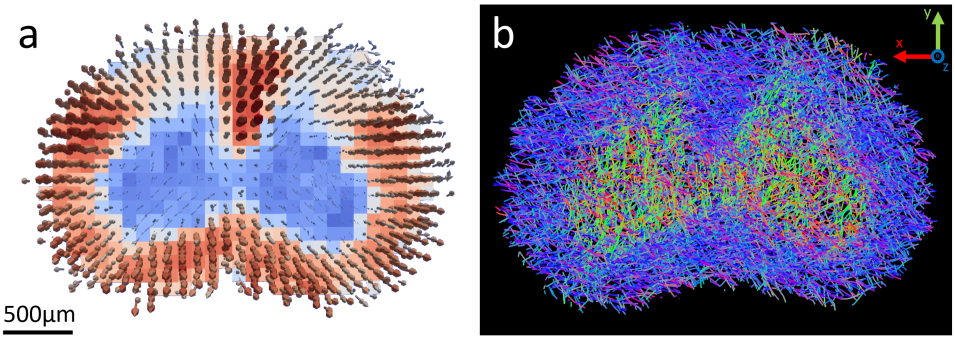

Supplementary Fig. 2. Diffusion-MRI based axon orientations and tracts for a virtual mouse spinal cord section.
a) Main axon orientations, derived as the eigenvector corresponding to the largest eigenvalue of the diffusion tensor, with the length weighted by fractional anisotropy. **b)** Probabilistic tractography based on the diffusion MRI dataset, with tracts colored based on arrow directions, similar to Fig. 3.

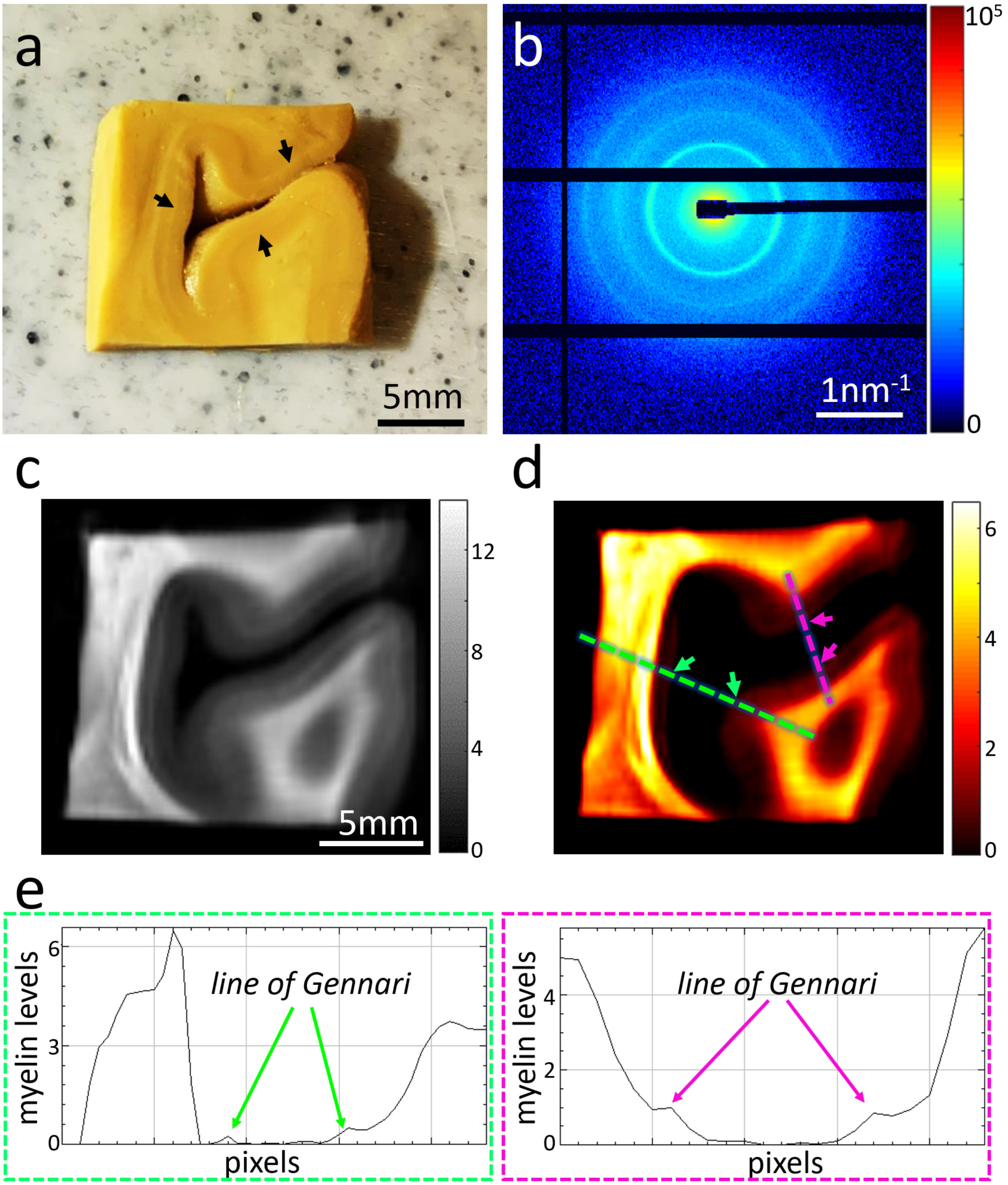

Supplementary Fig. 3. SAXS-TT on human primary visual cortex of a 78yo female.
a) The scanned specimen, with the line of Gennari pinpointed by the arrows. **b)** Characteristic diffraction pattern of the specimen, scanned in the 4-2 beamline of SLAC National Accelerator Laboratory. **c)** Virtual cross-section from the tensor tomographic reconstruction of the SAXS signal at the q-values of the myelin peak. **d)** Same virtual section depicting myelin levels from myelin-specific signal tensor tomographic reconstruction. **e)** Line plots across the green and magenta lines of (d), with line of Gennari indicated by arrows.

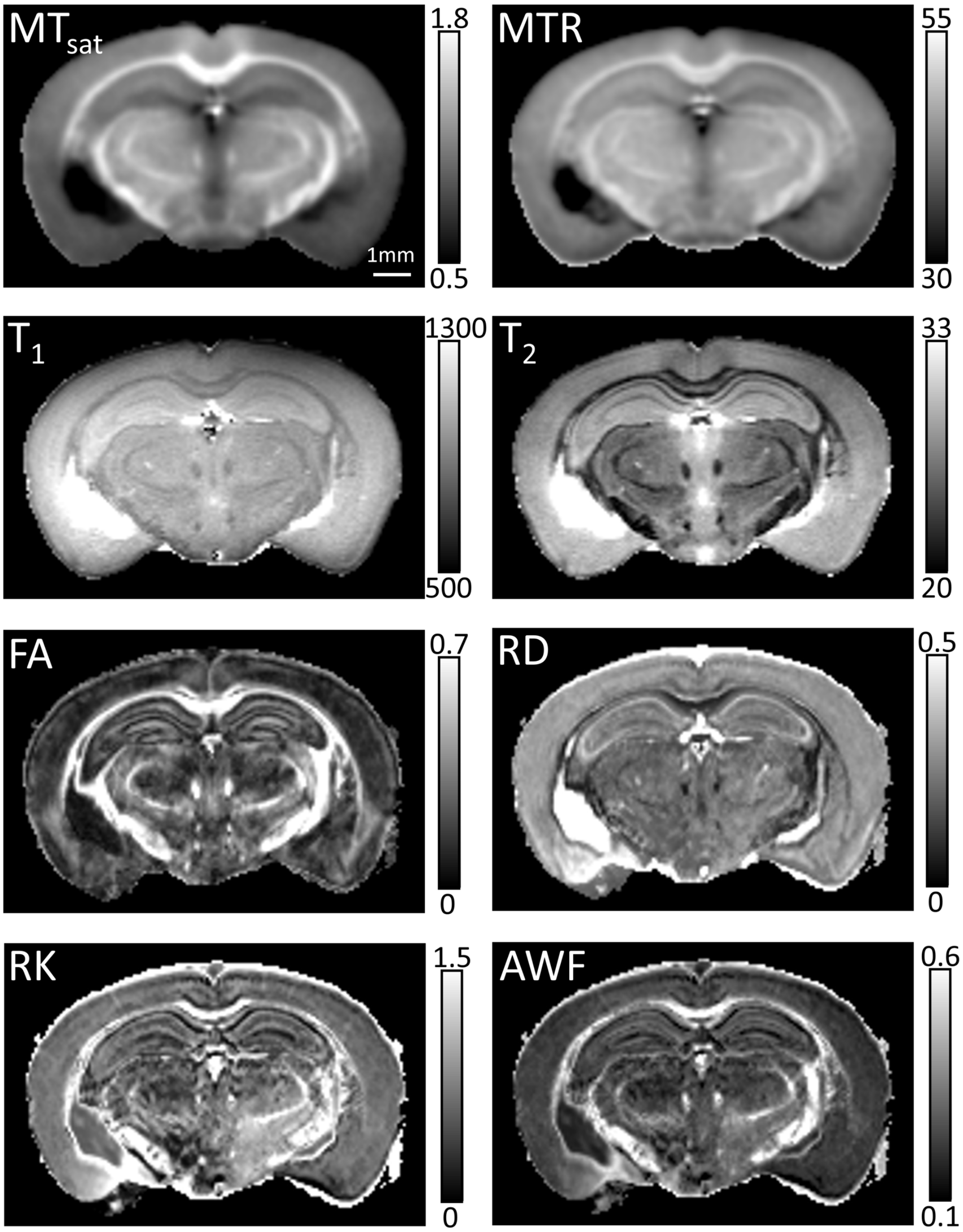

Supplementary Fig. 4. Coronal section of the MRI myelin-sensitive measures.
First row: magnetization transfer saturation (MT_{sat}),[27] and magnetization transfer ratio (MTR). Second row: T_1 and T_2 relaxation times maps. 3rd and 4th row: diffusion and kurtosis parameters[29] calculated by the DESIGNER pipeline.[48] FA: fractional anisotropy, RD: radial diffusivity, RK: radial kurtosis, AWF: axonal water fraction.

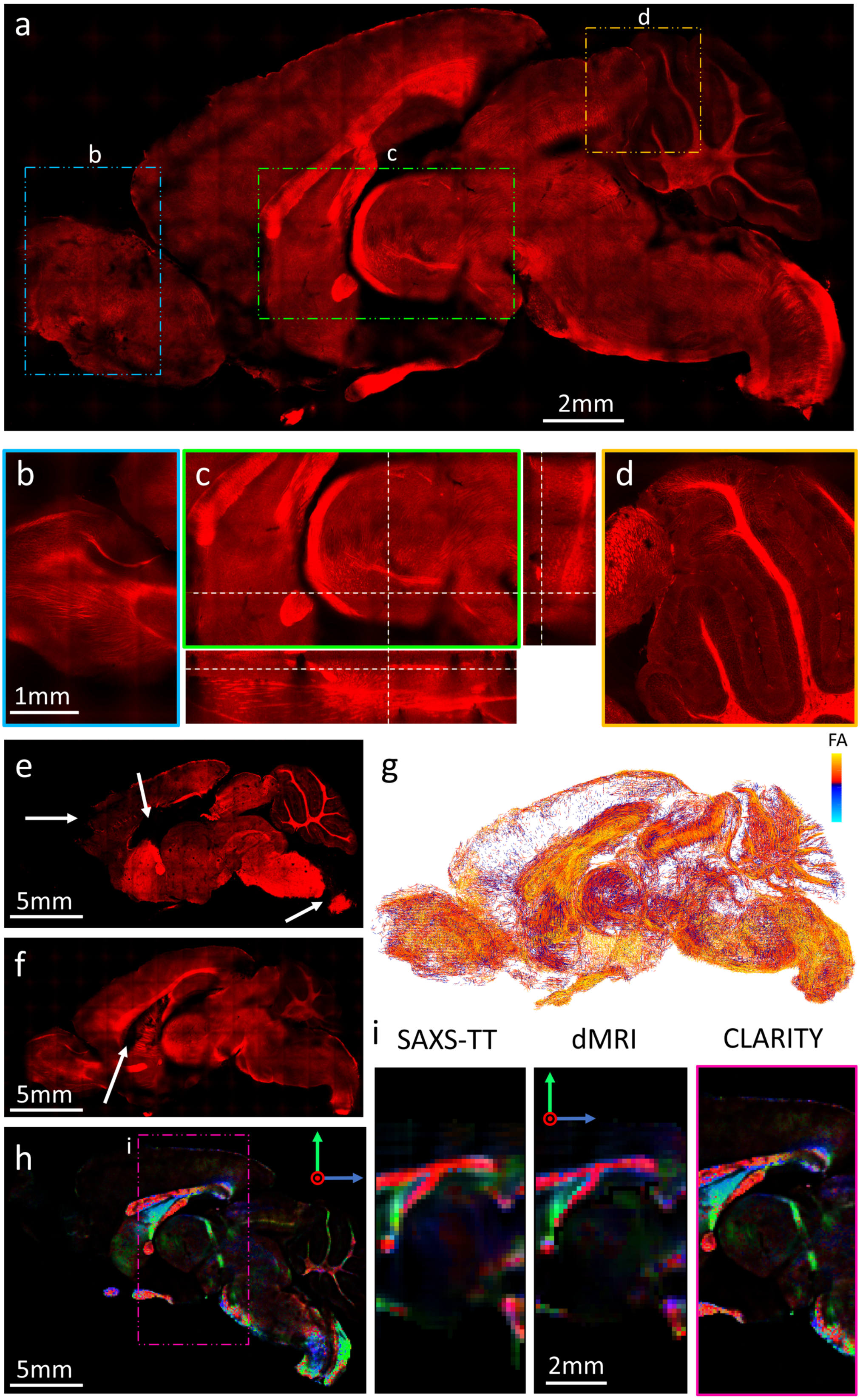

Supplementary Fig. 5. CLARITY-cleared and neurofilament-stained mouse brain hemisphere, previously scanned with diffusion MRI and SAXS-TT.
a) Sagittal virtual section, with the areas in **(b)**, **(c)** and **(d)** highlighted. (b) and (d) are from the same brain location at a different sagittal plane. (c) includes horizontal and coronal views at the positions of the dotted line. **e,f)** Sagittal sections at different depths, showing artifacts (white arrows) from inhomogeneous tissue expansion. **g)** Fiber orientations from structure tensor, scaled and colored by fractional anisotropy (FA). **h)** Color-encoded sagittal section. Color depicts fiber orientations according to reg-green-blue arrows, color intensity is weighted non-linearly by staining intensity (cf. Methods). Box encloses area for (i). **i)** Same area for the SAXS-TT, dMRI and CLARITY datasets, depicting color-encoded fiber directions.

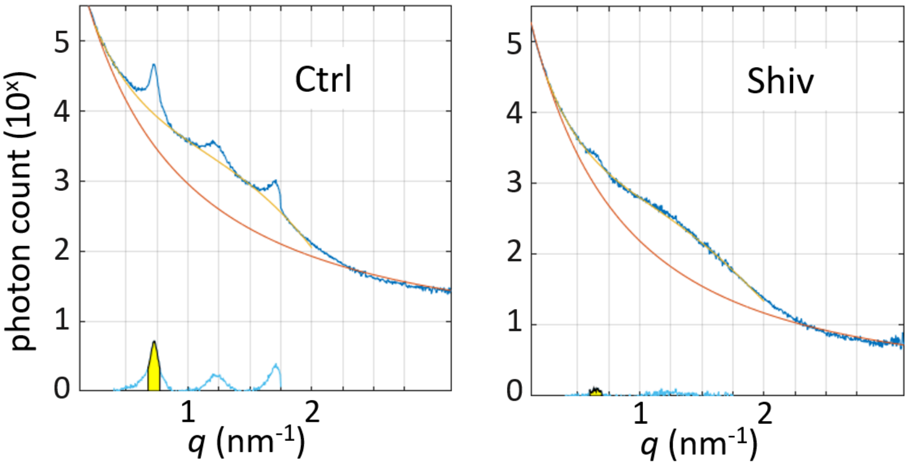

Supplementary Fig. 6. Myelinated *vs*. shiverer CNS SAXS signal.
Left: SAXS signal through corpus callosum of normally myelinated mouse. **Right:** SAXS signal through corpus callosum of shiverer mouse brain. Myelin peak for shiverer is much weaker, and at lower q-values ($q \approx 0.68 nm^{-1}$), corresponding to a “looser” myelin wrapping ($d_{CNS} \approx 18.5 nm$).

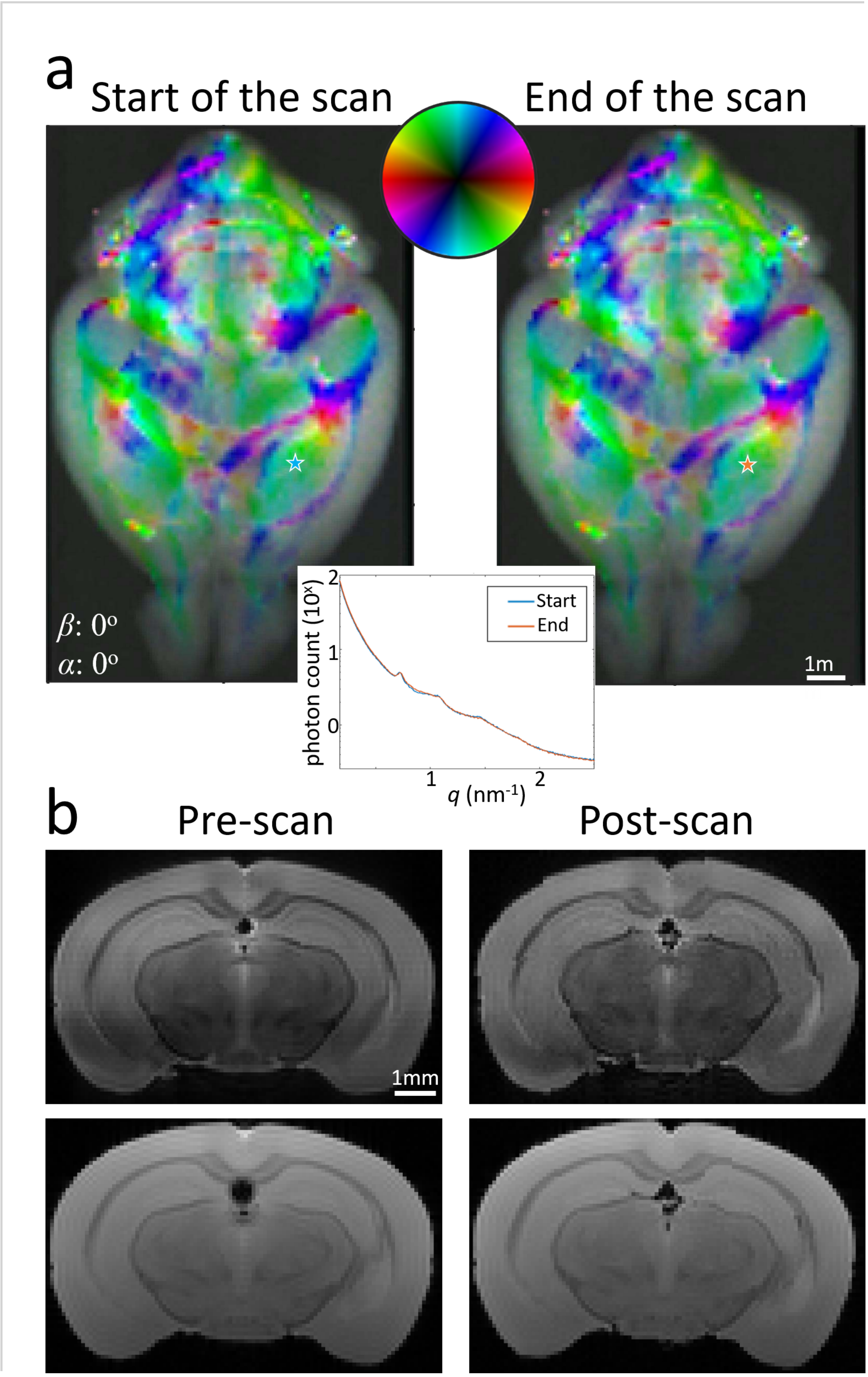

Supplementary Fig. 7. X-ray and MRI data before and after the SAXS-TT scan of the C57BL/6 mouse brain sample.
a) SAXS projections for sample orientation (β, α) = (0°,0°) at the beginning (first projection -left) and the end (after the last projection -right) of the SAXS-TT scan. Inset: Scattering intensity-q plot for the same sample point in the beginning and the end of the scan (marked by a star in the projection figures).
b) T_2- and T_1-weighted MRI scans (top and bottom row respectively) acquired a few days before (left) and after (right) the SAXS-TT scan.

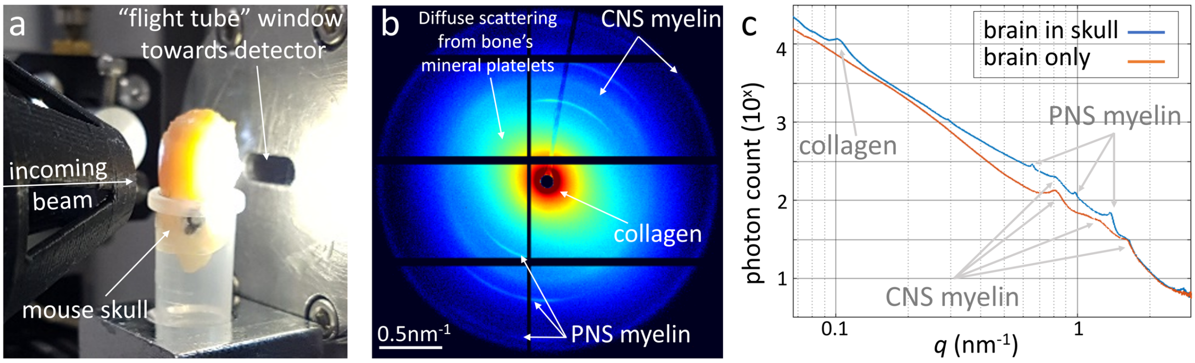

Supplementary Fig. 8. Feasibility of scanning the brain within the skull.
a) Mouse skull placed on a container, and shortly scanned. **b)** Diffraction pattern from the brain within the skull, with the CNS and PNS myelin peaks clearly visible, the collagen peak appearing at lower angles/q-values, and a significant diffuse scattering contribution from the hydroxyapatite mineral platelets present in skull’s bone tissue. **c)** Comparison of scattering intensities from the brain within (blue) and outside of the skull (orange), at approximately the same point. Myelin peaks are present in both curves. PNS nerves were severed during the brain extraction process and thus not appear in the brain-only case.

References

56. DiFabio, J. *et al.* The life science x-ray scattering beamline at NSLS-II. *AIP Conf. Proc.* **1741**, 30049 (2016).

57. Henrich, B. *et al.* PILATUS: A single photon counting pixel detector for X-ray applications. *Nucl. Instruments Methods Phys. Res. Sect. a-Accelerators Spectrometers Detect. Assoc. Equip.* **607**, 247–249 (2009).
