# Supplementary material for: Nanostructure-specific X-ray tomography reveals myelin levels, integrity and axon orientations in mouse and human nervous tissue
Source: Nat Commun. 2021 May 19;12:2941. doi: 10.1038/s41467-021-22719-7 (PMC8134484; doi:10.1038/s41467-021-22719-7)
Supplement: Supplementary file 3 — Description of Additional Supplementary Files [file 41467_2021_22719_MOESM3_ESM.pdf]

SUPPLEMENTARY MOVIES

Supplementary Movie 1. X-ray scattering patterns along a trajectory across a mouse brain. Displayed SAXS scattering patterns are from the C57BL/6 mouse brain, which was embedded in a PBS-based agarose gel, within a Kapton tube.

Supplementary Movie 2. All 267 SAXS-TT projections of the C57BL/6 mouse brain. Left: 2D projection image of C57BL/6 mouse brain sample embedded in a PBS-based agarose gel, upon variation of azimuth and elevation angles (α, β). Colors indicate in-plane fiber orientation, see colorwheel for reference. Right: Corresponding X-ray transmission image.

Supplementary Movie 3. All 254 SAXS-TT projections of the human corpus callosum splenium. The white matter sample is SAXS-TT scanned upon variation of azimuth and elevation angles (α, β). Colors indicate in-plane fiber direction, see colorwheel for reference. Most projections display little heterogeneity in axon orientation, due to the sample's homogeneity in fiber orientations. For few projections, a more complex in-plane orientation pattern appears. In these cases, the primary fiber orientation is aligned along the beam, which allows the secondary anisotropy (along the minor axes) in the structure to be probed.

Supplementary Movie 4. Extracting myelin-specific information from raw data across mouse brain. Fitting and myelin-specific information isolation is shown for each diffraction pattern segment, at the position of the 2nd-order myelin peak (q≈0.75nm^{-1}) along a trajectory across the Rag2$^{-/-}$ mouse brain for sample orientation (β, α) = (0,0). Compared to Fig. 6b and Supplementary Fig. 6, the intermediate fitting step is also shown here, with the signal as green line and the polynomial fit in magenta.
