# Supplementary material for: Nanostructure-specific X-ray tomography reveals myelin levels, integrity and axon orientations in mouse and human nervous tissue
Source: Nat Commun. 2021 May 19;12:2941. doi: 10.1038/s41467-021-22719-7 (PMC8134484; doi:10.1038/s41467-021-22719-7)
Supplement: Supplementary file 8 — Reporting Summary [file 41467_2021_22719_MOESM8_ESM.pdf]

nature research

Corresponding author(s): Marios Georgiadis
Last updated by author(s): Feb 19, 2021

Reporting Summary

Nature Research wishes to improve the reproducibility of the work that we publish. This form provides structure for consistency and transparency in reporting. For further information on Nature Research policies, seeAuthors & Referees and theEditorial Policy Checklist .

Statistics

For all statistical analyses, confirm that the following items are present in the figure legend, table legend, main text, or Methods section.

n/a	Confirmed	
☐	☒	The exact sample size (*n*) for each experimental group/condition, given as a discrete number and unit of measurement
☒	☐	A statement on whether measurements were taken from distinct samples or whether the same sample was measured repeatedly
☐	☒	The statistical test(s) used AND whether they are one- or two-sided<br>*Only common tests should be described solely by name; describe more complex techniques in the Methods section.*
☒	☐	A description of all covariates tested
☐	☒	A description of any assumptions or corrections, such as tests of normality and adjustment for multiple comparisons
☐	☒	A full description of the statistical parameters including central tendency (e.g. means) or other basic estimates (e.g. regression coefficient) AND variation (e.g. standard deviation) or associated estimates of uncertainty (e.g. confidence intervals)
☐	☒	For null hypothesis testing, the test statistic (e.g. *F*, *t*, *r*) with confidence intervals, effect sizes, degrees of freedom and *P* value noted<br>*Give P values as exact values whenever suitable.*
☒	☐	For Bayesian analysis, information on the choice of priors and Markov chain Monte Carlo settings
☒	☐	For hierarchical and complex designs, identification of the appropriate level for tests and full reporting of outcomes
☐	☒	Estimates of effect sizes (e.g. Cohen's *d*, Pearson's *r*), indicating how they were calculated

Our web collection on statistics for biologists contains articles on many of the points above.

Software and code

Policy information about availability of computer code

Data collection	no software was used for data collection
Data analysis	All analyses were performed in and plots were generated with Matlab 2016b,2017b,2018b,2019b,2020b.<br>Connectivity was derived using tckgen (MRtrix3), and mrview (MRtrix3) was used for visualizing resulting tracts.<br>FIJI (ImageJ1.52p) was used to stitch, correct and visualize the CLARITY dataset, and to register and visualize myelin histology images.<br>Paraview 5.7.0 was used for creating the 3D renderings.<br>SAXS data handling has been done using the "Base package", Scanning SAXS package", and "SASTT package" of the cSAXS beamline, Swiss Light Source, found at https://www.psi.ch/en/sls/csaxs/software.<br>IRTT reconstruction code can be found @ https://zenodo.org/record/1480589#.YBefZ2RKjAy.<br>The DESIGNER pipeline is @ https://github.com/NYU-DiffusionMRI/DESIGNER.<br>The myelin-specific signal extraction code (and demo dataset) has been deposited @ 10.5281/zenodo.4496831.

For manuscripts utilizing custom algorithms or software that are central to the research but not yet described in published literature, software must be made available to editors/reviewers. We strongly encourage code deposition in a community repository (e.g. GitHub). See the Nature Research guidelines for submitting code & software for further information.

Data

Policy information about availability of data

All manuscripts must include a data availability statement. This statement should provide the following information, where applicable:

- Accession codes, unique identifiers, or web links for publicly available datasets
- A list of figures that have associated raw data
- A description of any restrictions on data availability

The SAXS data that support the findings of this study are available in [repository name e.g "figshare"] with the identifier(s) [data DOI(s) e.g. "doi:10.6084/m9.figshare.1499292_D8"]

Field-specific reporting

Please select the one below that is the best fit for your research. If you are not sure, read the appropriate sections before making your selection.

☒ Life sciences ☐ Behavioural & social sciences ☐ Ecological, evolutionary & environmental sciences

For a reference copy of the document with all sections, see nature.com/documents/nr-reporting-summary-flat.pdf

Life sciences study design

All studies must disclose on these points even when the disclosure is negative.

Sample size	This study includes proof-of-principle experiments and feasibility demonstrations. We used:<br>one mouse brain, one mouse spinal cord, two human white matter and one cortex specimen for proof-of-principle experiments, two mouse brains (one control and one dysmyelinated) for demonstration of sensitivity of the method to myelin levels and nanostructural alterations
Data exclusions	no data were excluded
Replication	These proof of principle experiments do not aim to show population differences, but demonstrate the capabilities of the method. These were shown in multiple samples and sample ROIs.
Randomization	Study contains proof of principle experiments. Samples were chosen randomly.
Blinding	These proof of principle experiments do not aim to show population differences, but demonstrate the capabilities of the method.

Reporting for specific materials, systems and methods

We require information from authors about some types of materials, experimental systems and methods used in many studies. Here, indicate whether each material, system or method listed is relevant to your study. If you are not sure if a list item applies to your research, read the appropriate section before selecting a response.

Materials & experimental systems

n/a	Involved in the study	
☐	☒	Antibodies
☒	☐	Eukaryotic cell lines
☒	☐	Palaeontology
☐	☒	Animals and other organisms
☐	☒	Human research participants
☒	☐	Clinical data

Methods

n/a	Involved in the study	
☒	☐	ChIP-seq
☒	☐	Flow cytometry
☐	☒	MRI-based neuroimaging

Antibodies

Antibodies used	primary:<br>chicken polyclonal anti-neurofilament heavy polypeptide IgY antibody, ab4680, lot: GR3241438-5, Abcam PLC, UK<br><br>secondary:<br>Alexa Fluor® 647 AffiniPure F(ab')$_2$ Fragment Donkey Anti-Chicken IgY (IgG) (H+L), from Jackson ImmunoResearch Inc., USA, RRID: AB_2340379, Code: 703-605-155
Validation	The primary antibody has been validated in hundreds of research studies, that can be found in the company's product webpage https://www.abcam.com/neurofilament-heavy-polypeptide-antibody-ab4680.html

Animals and other organisms

Policy information about studies involving animals; ARRIVE guidelines recommended for reporting animal research

Laboratory animals	one 5-month-old C57BL/6 female mouse, one 50-day-old Rag2-/- male mouse, one 50-day-old Rag2-/-sh-/- male mouse<br>Housing conditions are described in the manuscript.
Wild animals	the study did not involve wild animals
Field-collected samples	the study did not involve samples collected from the field
Ethics oversight	C57BL/6 mouse: animal license ZH242/14 of the Animal Imaging Center of ETH Zurich/University of Zurich<br>50-day-old Rag2-/- and Rag2-/-sh-/- mice: Johns Hopkins University animal care and use committee protocol number MO16M313

Note that full information on the approval of the study protocol must also be provided in the manuscript.

Human research participants

Policy information about studies involving human research participants

Population characteristics	1. White matter specimens from 24.9-month-old female, subject with no indication of neuropathology, from the Sudden and Unexpected Death in Children (SUDC) biobank of NYU Langone<br>2. Human cortex specimen from a formalin-fixed brain of a 78 year-old female with no pathological finding in the cortex, from the tissue bank of Stanford's Alzheimer's Disease Research Center (ADRC).
Recruitment	1. Brain was donated with written consent of the parents, in the absence of compensation<br>2. Brain was donated to Stanford's Alzheimer's Disease Research Center (ADRC) with written consent prior to death
Ethics oversight	1. NYU Langone Institutional Review Board decision for study i14-01061<br>2. Stanford University Human Subjects Research Institutional Review Board, protocol # 33727.

Note that full information on the approval of the study protocol must also be provided in the manuscript.

Magnetic resonance imaging

Experimental design

Design type	N/A: The study shows proof of principle experiments in single subjects/animals/samples.
Design specifications	N/A: The study shows proof of principle experiments in single subjects/animals/samples.
Behavioral performance measures	N/A: The study shows proof of principle experiments in single subjects/animals/samples.

Acquisition

Imaging type(s)	diffusion MRI, magnetization transfer, T1 and T2 mapping
Field strength	9.4T Bruker BioSpec 94/30 and 7T Bruker 7030 Biospec
Sequence & imaging parameters	for the C57BL/6 mouse brain:<br>diffusion MRI:<br>segmented 3D SE-EPI (spin-echo echo planar imaging) sequence, echo time (TE) 42.9ms, repetition time (TR) 500ms, diffusion gradient duration δ=5.5ms, gradient separation Δ=12.1ms, isotropic voxel size 75µm, 2 averages, 173×126×211 (=12.975×9.45×15.825mm3) matrix<br>magnetization transfer:<br>FLASH (fast low angle shot) sequence, with TE=3.5ms, TR=400ms, 6 averages, isotropic voxel size 150µm, matrix 113×73×45 (=17×11×6.7mm3), MT pulse applied at 1,500 Hz offset with 40µT B1-amplitude<br>T1 mapping:<br>RAREVTR (Rapid Acquisition with Refocused Echoes and Variable TR) sequence, with TE=7.1ms, multiple TRs at [100, 200, 400, 800, 1,000, 1,200, 1,400, 1,600, 2,000, 3,000] ms, isotropic voxel size 150µm, matrix 226×147×110 (=17×11×8.25mm3)<br>T2 mapping:<br>MSME (Multi-Slice Multi-Echo) sequence, with TR=3,000ms, 25 TEs from 8.3 to 207.5ms every 8.3ms, isotropic voxel size 150µm, matrix 226×147×110 (=17×11×8.25mm3)<br>quantitative magnetization transfer:<br>proton-density-weighted (PDw), magnetization-transfer-weighted (MTw) and T1-weigthed (T1w) MGE (multi-gradient echo) scans at 14 TEs from 1.5ms to 14.5ms every 1ms, TR=25ms, flip angle: MTw,PDw=6o, T1w=15o, 20 averages, isotropic voxel size 150µm, matrix 155×133×59 (=23.25×19.95×8.85mm3). For the MTw scan, the MT pulse was applied at an offset of 3,000 Hz with a B1-amplitude of 10µT.

Rag2-/- mouse spinal cord:
diffusion MRI:
GRASE (GRAdient and Spin Echo) sequence, Echo time (TE) was 39.9ms, repetition time (TR) was 400ms, diffusion gradient duration was δ=8.5ms, gradient separation was Δ=17ms, with isotropic voxel size 100µm, matrix 128×104×120 (=12.8×10.4×12mm3)

Area of acquisition: whole-brain, cervical spinal cord (whole excised sample)

Diffusion MRI: ☒ Used ☐ Not used

Parameters: C57BL/6 mouse brain:
diffusion encoding was applied along 200 directions, of which 20 directions for b=1 ms/µm², 40 for 2 ms/µm², 60 for 3 ms/µm² and 80 for 4 ms/µm², along with 5 b=0 scans for each b-value.

Rag2-/- mouse spinal cord:
Diffusion encoding was applied along 170 directions, of which 5 directions for b-value=0.5 ms/µm², 10 for 1 ms/µm², 15 for 2.5 ms/µm², 20 for 5 ms/µm², 30 for 10 ms/µm², 40 for 15 ms/µm², and 50 for 20 ms/µm², along with 2 b=0 scans for each b-value.

Preprocessing

Preprocessing software: n/a: Data were retrieved from the scanner and post-processed with the DESIGNER pipeline mentioned below

Normalization: n/a: Data were retrieved from the scanner and post-processed with the DESIGNER pipeline mentioned below

Normalization template: n/a: Data were retrieved from the scanner and post-processed with the DESIGNER pipeline mentioned below

Noise and artifact removal: we used the DESIGNER pipeline (reference #49, https://github.com/NYU-DiffusionMRI/DESIGNER), which includes removal of noise and Gibbs artifacts, correction for inhomogeneities of the B1 field, as well as eddy current correction.

Volume censoring: n/a: Data were retrieved from the scanner and post-processed with the DESIGNER pipeline mentioned above

Statistical modeling & inference

Model type and settings: C57BL/6 mouse brain myelin level left-right hemisphere ROI analysis:
two-tailed unpaired t-test to compare myelin levels in all voxels of left-right ROIs

Human corpus callosum samples myelin level analysis:
two-tailed unpaired t-test to compare body and splenium myelin levels across all voxels

Effect(s) tested: whether voxel populations are derived from the same distribution

Specify type of analysis: ☐ Whole brain ☐ ROI-based ☒ Both

Anatomical location(s): C57BL/6 mouse brain myelin level left-right hemisphere ROI analysis:
A list of 21 ROIs in the mouse brain was arbitrarily chosen based on the author's perceived importance of the brain regions and their ability to cover a range of gray and white matter structures of the mouse brain.

Statistic type for inference (See Eklund et al. 2016): p-value

Correction: None

Models & analysis

n/a	Involved in the study	
☒	☐	Functional and/or effective connectivity
☒	☐	Graph analysis
☒	☐	Multivariate modeling or predictive analysis
