# Supplementary material for: Nanostructure-specific X-ray tomography reveals myelin levels, integrity and axon orientations in mouse and human nervous tissue
Source: Nat Commun. 2021 May 19;12:2941. doi: 10.1038/s41467-021-22719-7 (PMC8134484; doi:10.1038/s41467-021-22719-7)
Supplement: Supplementary file 9 — Source Data [file 41467_2021_22719_MOESM9_ESM.zip › Figure_Source_data_xls_Readme.rtf]

Files explanation:All files are spreadsheet (Microsoft Excel ‘.xlsx’) files‘Fig2_mouse_brain_ROIs.xlsx’Contains the myelin level values for all ROI voxels from the right and left hemispheres of the mouse brain. Right and left hemisphere data are in separate sheets in the file.‘Fig4_splenium_and_body_myelin_levels.xlsx’Contains the myelin level values for all human corpus callosum body and splenium voxels, in a column each.‘Fig5_histology_SAXS-TT_MRI_corrrelations.xlsx’Contains the values of all voxels from the MRI, SAXS-TT and histology parameter maps being compared.‘Fig7_ctrl_shiverer_myelin_levels.xlsx’Contains the myelin level values for all voxels in each region of interest for the control and Shiverer mouse brains. Control and shiverer data are in separate sheets in the file.
